# Sexual Dimorphism in Impairment of Acetylcholine-Mediated Vasorelaxation in Zucker Diabetic Fatty (ZDF) Rat Aorta: A Monogenic Model of Obesity-Induced Type 2 Diabetes

**DOI:** 10.3390/ijms252011328

**Published:** 2024-10-21

**Authors:** Rifat Ara Islam, Xiaoyuan Han, Sonali Shaligram, Mitra Esfandiarei, John N. Stallone, Roshanak Rahimian

**Affiliations:** 1 Department of Pharmaceutical Sciences, Thomas J. Long School of Pharmacy, University of the Pacific, Stockton, CA 95211, USA; r_islam2@u.pacific.edu (R.A.I.); sonali.shaligram@gmail.com (S.S.); 2 Department of Biomedical Sciences, Arthur A. Dugoni School of Dentistry, University of the Pacific, Stockton, CA 94115, USA; xhan@pacific.edu; 3 Biomedical Sciences Program, College of Graduate Studies, Midwestern University, Glendale, AZ 85308, USA; mesfan@midwestern.edu; 4 Department of Veterinary Physiology and Pharmacology and Michael E. DeBakey Institute for Comparative Cardiovascular Sciences, School of Veterinary Medicine, Texas A&M University, College Station, TX 77843-4466, USA; jstallone@cvm.tamu.edu

**Keywords:** type 2 diabetes (T2D), aortic vasorelaxation, sex differences, nitric oxide (NO), Zucker Diabetic Fatty (ZDF) rats

## Abstract

Several reports, including our previous studies, indicate that hyperglycemia and diabetes mellitus exert differential effects on vascular function in males and females. This study examines sex differences in the vascular effects of type 2 diabetes (T2D) in an established monogenic model of obesity-induced T2D, Zucker Diabetic Fatty (ZDF) rats. Acetylcholine (ACh) responses were assessed in phenylephrine pre-contracted rings before and after apocynin, a NADPH oxidase (NOX) inhibitor. The mRNA expressions of aortic endothelial NOS (eNOS), and key NOX isoforms were also measured. We demonstrated the following: (1) diabetes had contrasting effects on aortic vasorelaxation in ZDF rats, impairing relaxation to ACh in females while enhancing it in male ZDF rats; (2) inhibition of NOX, a major source of superoxide in vasculature, restored aortic vasorelaxation in female ZDF rats; and (3) eNOS and NOX4 mRNA expressions were elevated in female (but not male) ZDF rat aortas compared to their respective leans. This study highlights sexual dimorphism in ACh-mediated vasorelaxation in the aorta of ZDF rats, suggesting that superoxide may play a role in the impaired vasorelaxation observed in female ZDF rats.

## 1. Introduction

Type 2 diabetes (T2D) is a public health burden affecting over 34.2 million people in the USA [1] and predisposes patients to higher risk of cardiovascular diseases (CVD), the leading cause of death globally [2,3]. Premenopausal women exhibit a lower rate of CVD compared to men of the same age [4,5]. However, those with diabetes not only experience the loss of this sex-based cardiovascular protection, but also face a higher risk of CVD compared to diabetic men [6,7]. Several reports, including our previous studies, indicate that hyperglycemia and diabetes mellitus exert differential effects on vascular function in males and females [8,9,10]. We previously reported sextual dimorphism in the vascular dysfunction in both small and large arteries of streptozotocin-induced type 1 diabetic rats [11,12,13], as well as the UC Davis Type 2 Diabetes Mellitus (UCD-T2DM), a polygenic model of T2D rats [10,14]. It is yet to be determined whether these reported sex differences in aortic function can be generalized to other widely used preclinical diabetic models.

To comprehensively understand the pathogenesis of diabetes, rodent models, such as db/db mice, Goto-Kakizaki (GK) rats, Otsuka Long-Evans Tokushima fatty (OLETF) rats, Zucker Diabetic Fatty (ZDF) rats, and UCD-T2DM rats have been developed as preclinical animal models of T2D [15,16]. In this study, we use the ZDF rat model. ZDF rats are a well-established monogenic obesity model of T2D, which is derived from the Zucker fatty (ZF) rat carrying a mutation in the leptin receptor gene (Lepr) and represents metabolic syndrome with sustained and early-onset hyperglycemia and progression to β-cell death [15,17]. Here, we hypothesized that there are sex differences in the development of vascular dysfunction in the aorta of ZDF rats.

Endothelial dysfunction is an initial indicator of vascular disease related to diabetes [18,19], and it is defined by a diminished endothelium-dependent vasorelaxation (EDV) to agonists like acetylcholine (ACh), as well as a decrease in flow-mediated vasodilation [20,21]. EDV is typically utilized as a reliable measure to assess endothelial function in different pathological conditions. It relies on several endothelium-derived relaxing factors (EDRFs), including nitric oxide (NO), prostacyclin, and endothelium-derived hyperpolarization, commonly referred to as endothelium-derived hyperpolarizing factor (EDHF) [22]. In conduit arteries such as aorta, NO is essential for regulating vascular responses under physiological conditions [23,24]. It is generally recognized that NO levels decrease in diabetes [25,26] and that alterations in superoxide and endothelial NO synthase (eNOS) may play a role in this decline in NO production [27]. Other investigators have shown both an increase [28,29] and a decrease [30,31] in eNOS expression in the aorta of diabetic rats. Thus, we aimed to assess the level of eNOS expression in aortas of male and female ZDF rats. We also examined whether inhibiting NADPH oxidase (NOX), a major cellular source of superoxide in the vascular system [32,33], would impact aortic relaxation in this model. Additionally, the mRNA expression levels of NOX isoforms (NOX1, NOX2, and NOX4) were analyzed in aortic tissues from male and female ZDF rats, as well as their non-diabetic (lean) controls.

## 2. Results

### 2.1. Assessments of Body Weight, Blood Glucose, and Insulin Resistance (IR) in ZDF Rats

At sixteen weeks of age, the body weights of ZDF rats were higher than those of lean rats, irrespective of their sex (Table 1). However, males had higher bodyweight compared to their female counterparts. The non-fasting blood glucose levels of female and male ZDF rats were significantly higher than those of their non-diabetic lean counterparts (Table 1). Plasma insulin levels were elevated, but did not reach a significant level (*p* = 0.06) in ZDF rats compared to their respective controls, regardless of sex (Table 1). To quantify insulin resistance (IR), HOMA-IR was calculated using blood glucose and plasma insulin levels. IR was significantly higher in male and female ZDF rats compared to their respective lean animals (Table 1).

### 2.2. Measurements of ACh-Induced Relaxation in ZDF Rat Aorta

**Figure 1:**
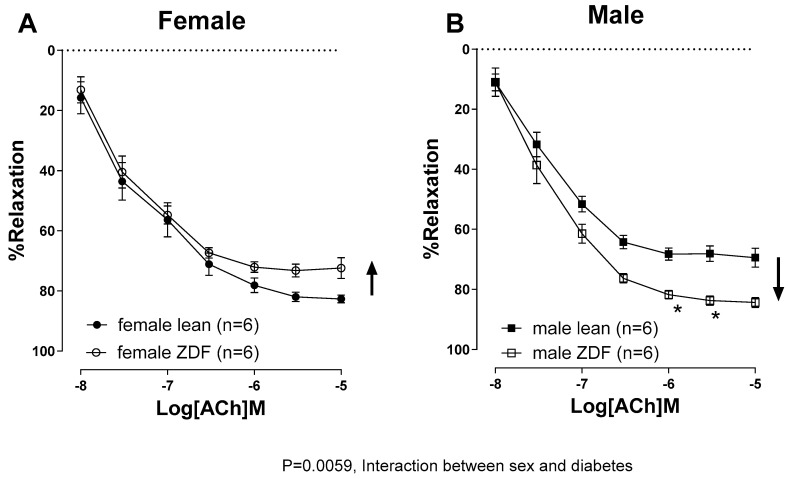
Acetylcholine (ACh, 10^−8^ to 10^−5^ M)-induced vasorelaxation in phenylephrine pre-contracted aortic rings from female (**A**) and male (**B**) lean and ZDF rats. Data are presented as mean ± SEM. *n* = 6 per group, analyzed using a three-way ANOVA followed by Tukey’s post hoc test. * *p* < 0.05 vs. male lean at the corresponding concentration of ACh.

The aortic rings taken from female lean rats exhibited a significantly greater vasorelaxation response to ACh compared to male lean rats (the E_max_ in female lean and male lean rats was 83 ± 1.3% and 71 ± 2.5%, respectively) (Table 2). In females, T2D attenuated the ACh-induced aortic relaxation; the E_max_ in female lean and female ZDF rats were 83 ± 1.3% and 74 ± 2.0%, respectively (Figure 1A, Table 2). However, in males, T2D significantly enhanced vasorelaxation to ACh; the E_max_ in male lean and male ZDF were 71 ± 2.5% and 84 ± 1.6%, respectively (Figure 1B, Table 2). The sensitivity of aortic rings to ACh, as measured using pD_2_, was not changed significantly in ZDF rats compared to their age-matched lean controls, regardless of sex (Table 2).

### 2.3. Effects of Apocynin on ACh-Induced Relaxation in ZDF Rat Aorta

**Figure 2:**
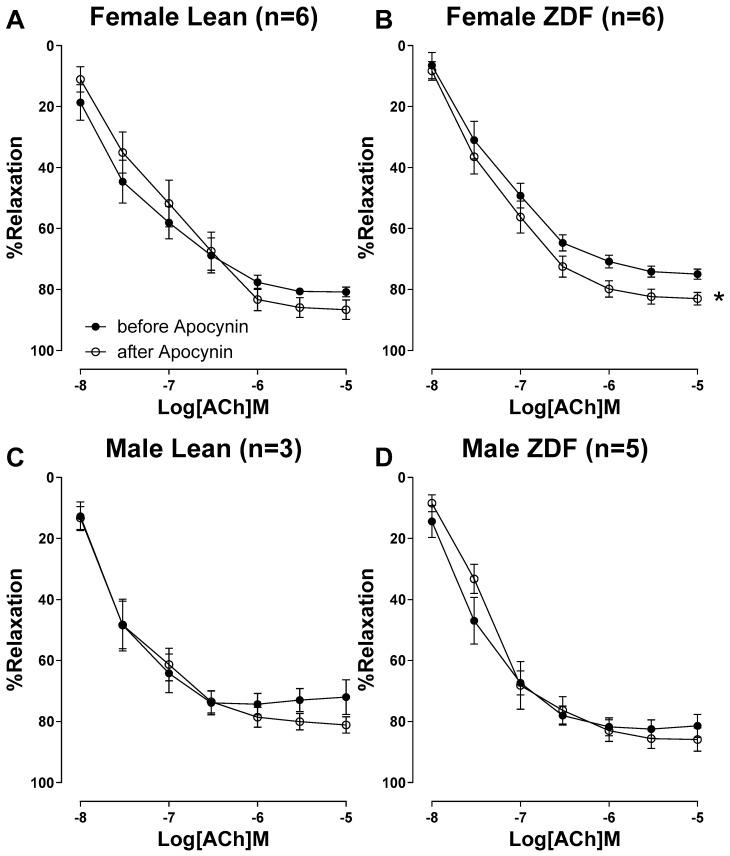
Acetylcholine (ACh, 10^−8^ to 10^−5^ M)-induced vasorelaxation in phenylephrine pre-contracted aortic rings from female lean (**A**), female ZDF (**B**), male lean (**C**), and male ZDF (**D**) rats. Relaxation to ACh was measured before and after incubation with Apocynin (100 μM), a NADPH oxidase inhibitor. Data are expressed as mean ± SEM. *n* = 3–6, analyzed using a two-way ANOVA with repeated measures. * *p* < 0.05 vs. before Apocynin.

Preincubation of aortas with apocynin (100 μM), a NOX inhibitor, did not change the ACh-induced relaxation in lean rats, irrespective of sex, as evidenced by the absence of significant differences in E_max_ or pD_2_ compared to the ACh response without the drug (although the lack of apocynin effect within male lean rats may be influenced by the small sample size). However, the concentration–response curve (CRC) to ACh was markedly enhanced after preincubation of aortic rings with apocynin in female ZDF, but not in male ZDF rats (Figure 2). Particularly, pretreatment of aorta with apocynin significantly enhanced the E_max_ to ACh only in female ZDF rats; the E_max_ to ACh in female ZDF rats was 75 ± 1.6% before apocynin and 83 ± 2.0% after apocynin (Table 3).

### 2.4. Measurements of Sodium Nitroprusside (SNP)-Induced Relaxation in ZDF Rat Aorta

**Figure 3:**
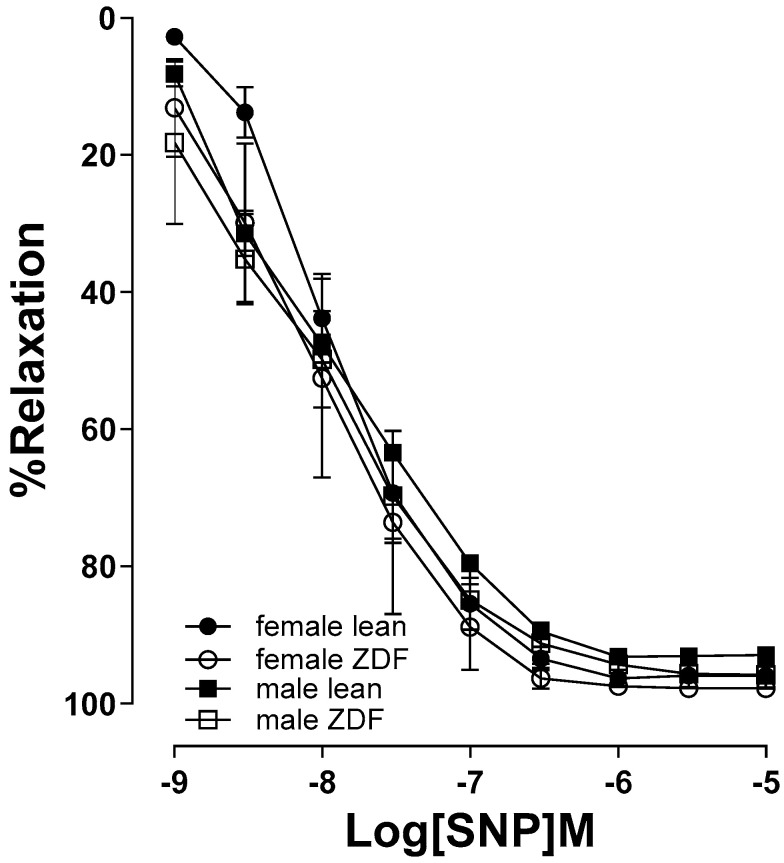
Sodium nitroprusside (SNP, 10^−9^ to 10^−5^ M)-induced vasorelaxation in phenylephrine pre-contracted aortic rings from male and female lean and ZDF rats. Data are expressed as mean ± SEM. *n* = 4–7, analyzed using a three-way ANOVA.

To determine whether the sensitivity of arteries to NO in male and female ZDF rats were affected, the CRC to SNP, a NO donor, was measured in PE (2 µM) pre-contracted aortic rings. Our data showed no significant statistical differences in CRC to SNP (10^−9^ to 10^−5^ M), between male and female leans, or male and female ZDF animals (Figure 3).

### 2.5. Assessments of PE-Induced Contractile Responses in ZDF Rat Aorta

To determine whether the contractile responses in male and female ZDF rats were affected, CRC to PE (10^−8^ to 10^−5^ M) in aortic rings were generated. The sensitivity and maximal contractile force developed in response to PE (Tension_max_) were not different between the sexes in lean or ZDF groups (Table 4).

**Figure 4:**
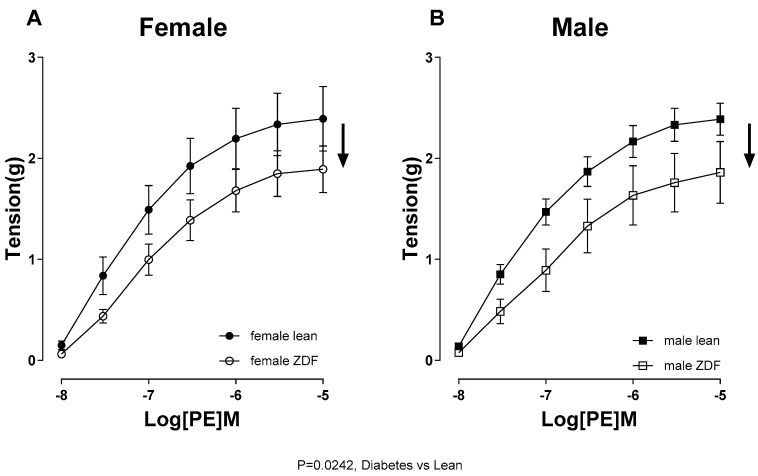
Phenylephrine (PE, 10^−8^ to 10^−5^ M)-induced vasoconstriction in aortic rings from female (**A**) and male (**B**) lean and ZDF rats. Data are expressed as mean ± SEM. *n* = 7 per group, analyzed using a three-way ANOVA followed by Tukey’s post hoc test.

**Figure 5:**
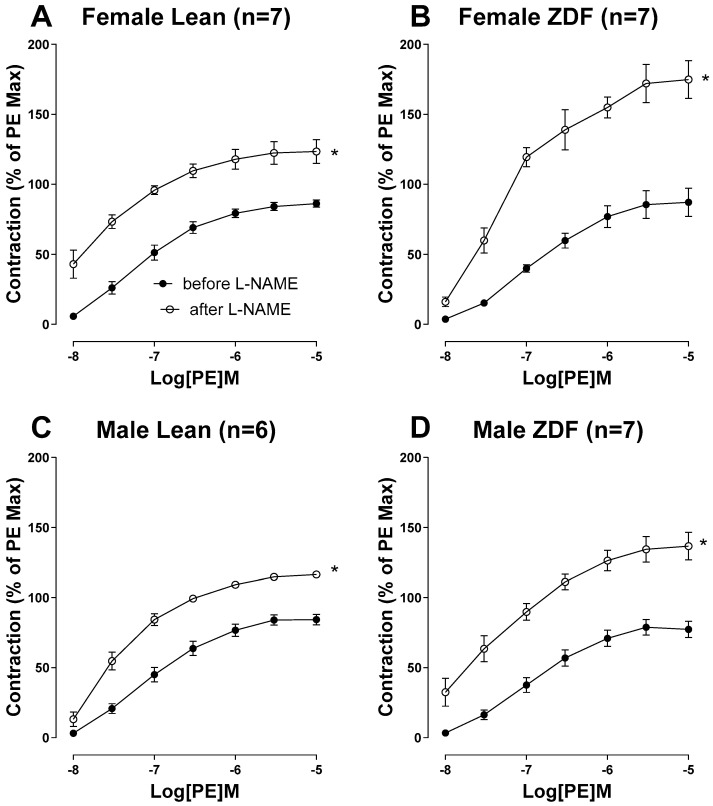
Phenylephrine (PE, 10^−8^ to 10^−5^ M)-induced vasoconstriction in aortic rings from female lean (**A**), female ZDF (**B**), male lean (**C**), and male ZDF (**D**) rats. Contractile responses to PE were generated before and after the addition of L-NAME (200 μM) in the presence of indomethacin (Indo, 10 µM). Data are expressed as mean ± SE. *n* = 6–7, analyzed using a two-way ANOVA with repeated measures. * *p* < 0.05 vs. before L-NAME in all groups.

However, ZDF rats displayed diminished CRC to PE in their aortas compared with their respective lean subjects, regardless of sex (Figure 4, diabetes vs. leans, *p* < 0.05). Next, we assessed the CRC to PE (10^−8^ to 10^−5^ M) in aortic rings before and after pretreatment with the NO synthase (NOS) inhibitor, L-NAME (200 µM), in the presence of indomethacin (10 µM). The administration of indomethacin to block cyclooxygenase (COX) metabolites marginally reduced the PE-induced contraction in aortas Significant alterations in the PE contractile response following the addition of L-NAME would elucidate the role of endothelium-derived NO during smooth muscle contraction, a phenomenon reported by us [10,34] and others [35,36]. Incubating the aortic rings with L-NAME led to a notable increase in the PE contractile responses across all four groups (Figure 5). However, as shown by ΔAUC, aortas from female ZDF rats significantly exhibited a more pronounced enhancement of the PE response after inhibition of NOS compared to their lean counterparts and male ZDF rats (Table 5).

### 2.6. Measurements of eNOS and NOX mRNA Expressions in ZDF Rat Aorta

**Figure 6:**
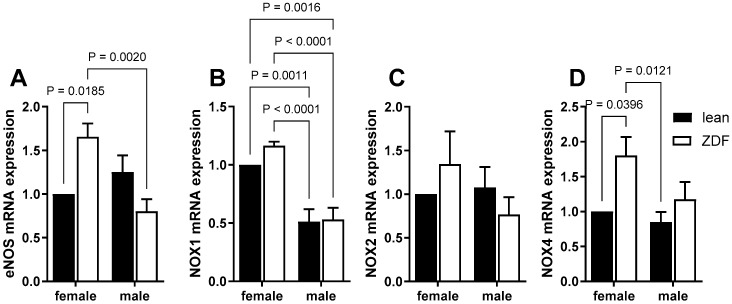
eNOS (**A**), NOX1 (**B**), NOX2 (**C**), and NOX4 (**D**) mRNA expression in male and female lean and ZDF rat aortas. Data are presented as mean ± SEM. *n* = 6, the mRNA expression levels of control lean female rats were arbitrarily normalized and set as one. Statistical differences (*p* < 0.05) between two groups are denoted by capped lines, analyzed using a two-way ANOVA followed by Tukey’s post hoc test.

To investigate a mechanism by which the vasorelaxation might be altered in ZDF rats, the mRNA expression of eNOS and key NOX isoforms in the aorta were determined using real-time RT-PCR. Intriguingly, the results showed a significant increase in eNOS mRNA expression in the aortas of female ZDF rats compared to their lean counterparts and male ZDF animals (Figure 6A). Next, we measured the mRNA expression levels of NOX isoforms (NOX1, NOX2, and NOX4), major sources of superoxide in the vascular wall. The mRNA expression level for NOX1 was significantly higher in female rat aortas (in both leans and ZDF) compared to male tissues (Figure 6B). As depicted in Figure 6C, there were no differences in NOX2 mRNA expression among the experimental groups of animals. However, the NOX4 mRNA expression level was significantly elevated only in female ZDF rats compared to their lean counterparts (Figure 6D).

## 3. Discussion

This study examines the effects of T2D on aortic reactivity in male and female rats of an established monogenic model of obesity-induced T2D, ZDF rats. Our findings indicate that the ACh-induced relaxation in female ZDF rat aortas is impaired, while, intriguingly, male aortas demonstrate enhanced vasorelaxation. Our data also suggest that the superoxide might play a role in the impaired vasorelaxation observed in female ZDF rats.

In T2D, studies have reported impaired [37], enhanced [38], or preserved [39] EDV. In this study, there was a notable reduction in the maximal relaxation response to ACh in aortic rings derived from female ZDF rats compared to their lean counterparts. Yet, this study’s intriguing finding was the observed enhancement in EDV in aortic rings from the age-matched male ZDF animals when compared to their respective lean controls. Similar observations were reported by our group in UCD-T2DM male and female rats [10]. On the other hand, decreased EDV was reported in 28–40-week-old male ZDF rats [40]. However, Oltman et al. did not find any change in EDV in 8–12-week-old or 16–24-week-old male ZDF rat aortas [40]. Moreover, no difference in EDV was observed in 14-week-old female ZDF rat aortas compared to their lean counterparts [41]. The source of these discrepancies is unclear. Factors such as the duration of disease and age, among others, may influence the alteration of vascular reactivity to vasodilators or vasoconstrictors in diabetes.

In the current study, at 16 weeks of age, both male and female ZDF rats displayed elevated body weight, blood glucose, insulin, and IR compared to their lean counterparts of the same age. Female ZDF rats had significantly lower body weight than male ZDF rats. In this study, the visceral fat and triglyceride levels in male and female ZDF rats were not measured. However, Corsetti et al. [42], who studied the development of diabetes in male and female ZDF rats, found that the characteristics of circulating lipoprotein particles in females were not influenced by diabetic conditions. They observed that females had a higher abundance of triglyceride-rich lipoprotein particles compared to the males [42]. As a result, we cannot rule out the possibility that elevated levels of triglyceride-enriched lipoproteins, which have been previously reported in ZDF rats, may contribute to the impaired vascular function observed in female ZDF rats.

It is widely established that NO serves as a primary mediator of EDV in conduit arteries [23,24]. The impairment of EDV in female ZDF rats observed in the current study may stem from elevated NO inactivation by reactive oxygen species (ROS) and/or its interaction with aortic smooth muscle. In the context of diabetes, research indicates that the elevated production of superoxide is a significant factor in diminishing EDV responses [43]. One major source of cellular stress is superoxide, which is primarily generated in the mitochondria and by the NOX family [44]. In this study, our data show that incubation of aortic rings with apocynin, a NOX inhibitor, significantly restored ACh responses in female ZDF aortas, suggesting a potential role of superoxide in the impairment of EDV in this group. Accordingly, when we examined specific isoforms of NOX mRNA expression, the levels of NOX4 mRNA expression were significantly enhanced in female ZDF rat aortas compared to their lean counterparts. The NOX family consists of seven isoforms, where NOX4 is the most well-studied one [45]. NOX4 mRNA has been found to be a prevalent transcript in cultured aortic smooth muscle cells from both rat and humans [46,47]. Elevated NOX4 expression in the aortic wall and vascular smooth muscle cells, isolated from aging aortas, has been reported in connection with aortic stiffening and atherosclerosis in hypercholesterolemic mice [48]. Ding et al. demonstrated that the increase in oxidative stress within the vasculature of STZ-induced diabetic type 1 ApoE−/− mice was linked to elevated expressions of eNOS, NOX4, and superoxide dismutase (SOD) [49]. Along a similar line, we observed an increased level of eNOS mRNA expression in the aortas of female ZDF rats compared with their lean counterparts and male ZDF rats.

It is generally accepted that NO level is reduced in diabetes and that variations in eNOS levels may play a role in the reduction in NO production [25,26]. However, previous studies demonstrated both an increase [28,29] and a decrease [30,31] in eNOS expression in diabetic rat aortas. Elevated eNOS expression, accompanied by endothelial dysfunction, has been observed in the STZ-induced type 1 diabetic mice vasculature [50]. It has been reported that an increased expression of eNOS may not be inherently beneficial. Previous studies reported that eNOS overexpression in apoE-deficient mice results in accelerated atherogenesis [51,52]. Moreover, when eNOS becomes uncoupled, it can act similarly as NOX, leading to excessive production of superoxide [50,53]. Here, we did not directly measure superoxide levels or eNOS uncoupling. Nevertheless, consistent with our working hypothesis is the observation that preincubation of aortic rings with apocynin, an inhibitor of NOX, notably restored the ACh responses in female ZDF rats. Similarly, we observed increased expressions of NOX4 and eNOS mRNA in the aortas of female ZDF rats compared to lean rats. These increases could be harmful and might contribute to vascular dysfunction by leading to a higher production of superoxide.

In this study, we also demonstrated that mRNA levels of NOX1 were significantly increased in aortas from the female rats compared to the males, regardless of diabetic state. Clearly, the functional consequence of enhanced aortic NOX1 mRNA expression in females compared to males needs to be investigated. Nevertheless, these data are consistent with a study reporting that the NOX1 protein level was regulated in a sex-dependent manner in female mice aortas [54].

Changes in EDV to ACh in the aortas of ZDF rats could also be attributed to variations in smooth muscle responsiveness to NO or alterations in contractile responses. However, our data show that the relaxation responses to SNP, a NO donor, was not altered by diabetes or sex. Previous studies, including our report, have also shown an altered EDV while endothelium-independent relaxation remains intact in diabetic models [10,55]. Furthermore, here we showed that there was no difference in the sensitivity to contractile agent among the experimental groups. However, the CRC to PE were significantly attenuated in aortic rings of ZDF groups of both sexes compared with their respective lean subjects. The decreased CRC to PE may, in part, explain the elevated maximal relaxation in ACh responses in aorta of male ZDF rats. However, it is crucial to consider that despite the decrease in PE contractile response, the ACh vasorelaxation was still impaired in female ZDF arteries. This indicates that the increased PE contractile responsiveness cannot be considered the reason for the diminished ACh responses seen in these arteries. Along a similar line, Oltman et al. [40] has shown that aortas from 8 to 12-week-old male ZDF rats exhibited attenuated maximal PE-induced constriction when compared with those from the lean animals (they did not examine females in their study).

Next, we assessed the level of endothelium-derived NO in response to PE in ZDF rat aortas by assessing the differences in the extent of PE-induced contraction with and without L-NAME [10,12,35,36]. Intriguingly, L-NAME produced a significantly greater potentiation of the PE response in aortic rings from female ZDF compared with those in lean counterparts and male ZDF rats. While the functional consequences of augmented NO in response to PE in the aortic rings of female ZDF rats are unclear, we previously reported a similar observation (a greater potentiation of the PE response by L-NAME) in female STZ-diabetic rat mesenteric arteries compared with diabetic male arteries [11]. Furthermore, it is important to point out that in these studies, we used L-NAME to inhibit NOS, which is a non-selective NOS inhibitor, and it can also block the uncoupled actions of NOS as well as inducible NOS (iNOS). Thus, the increased L-NAME responses observed in the arteries of female ZDF rats may involve not only uncoupled eNOS, a main source of vascular superoxide in diabetes [56], but also iNOS. Along similar lines, Bardell and MacLeod [57] reported that the elevated L-NAME responses in diabetic arteries may implicate iNOS in vascular smooth muscle, and this could increase superoxide, particularly in conditions where peroxynitrite formation is more prevalent [53].

In conclusion, this study highlights sexual dimorphism in ACh-mediated vasorelaxation in the aortas of ZDF rats, a monogenic T2D model. Further investigation of the mechanisms underlying sex differences in vascular relaxation in this T2D model could help identify sex-specific targets, such as the distinct role of superoxide. The knowledge gained will ultimately (1) improve our understanding of the mechanisms driving sex-specific vascular dysfunction in diabetes, (2) clarify how biological sex influences vascular diseases in experimental diabetic models, and (3) provide insights for developing targeted therapeutic strategies to reduce cardiovascular complications. Eventually, findings from preclinical studies, followed by clinical investigations, will determine whether biological sex could affect treatment outcomes and guide the development of more effective diabetes management strategies that consider these differences.

**Figure 7:**
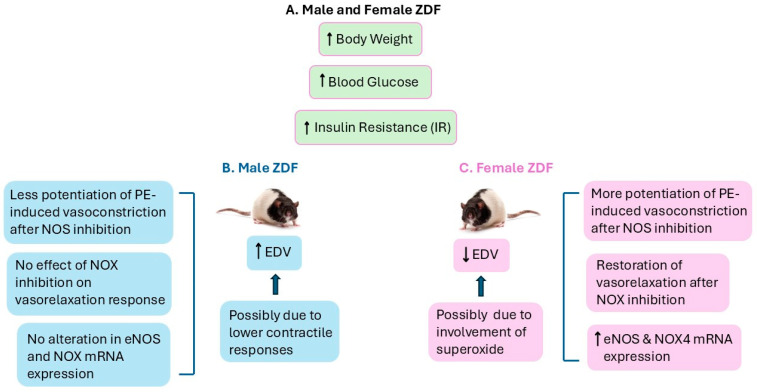
Proposed scheme based on the data presented in this study. Both ZDF male and female rats exhibited increased body weight, elevated glucose levels, and insulin resistance (IR) ((**A**) green one). EDV was increased in the aortic rings from ZDF male rats, possibly due to enhanced contractile responses ((**B**) blue one), but was impaired in female ZDF rats, possibly due to the elevated involvement of superoxide ((**C**) pink one). EDV, endothelium-dependent vasorelaxation; PE, phenylephrine; NOS, nitric oxide synthase; eNOS, endothelial nitric oxide synthase; NOX4, NADPH oxidase 4; ↑ Increase, ↓ decrease.

Figure 7 describes our proposed framework based on the data provided in this study. Both male and female ZDF exhibited increased body weight, elevated glucose levels, and insulin resistance (IR) (Figure 7A). Endothelium-dependent vasorelaxation (EDV) was enhanced in aortic rings from male ZDF rats (Figure 7B) but was impaired in female ZDF rats (Figure 7C). In male ZDF rats, the increased EDV was accompanied by less potentiation in PE-induced contraction after NOS inhibition, no effect of NOX inhibition on vasorelaxation response, and no alteration in eNOS and NOX4 mRNA expression, and the increased EDV in this group could, in part, be attributed to the decreased contractile responses to PE. However, in female ZDF rats, the decreased EDV was accompanied with a greater potentiation of PE-induced vasocontraction after NOS inhibition, restoration of vasorelaxation after NOX inhibition, and elevated eNOS and NOX4 mRNA expression, and the decreased EDV in this group could, in part, be attributed to the potential involvement of superoxide.

### Limitations

Although we did not measure the sex hormones in this model, alterations in circulating sex hormone levels (estrogen and androgen) in diabetes have been well-documented [58,59,60,61]. For example, reduced estrogen levels in both type 1 and type 2 diabetic female rats have been reported by other investigators [58]. Therefore, one potential explanation for the sex differences observed in our study is that the reduced circulating estrogen levels in female ZDF rats may have contributed to the impaired NO-mediated vasorelaxation in this group. However, it is important to note that the primary objective of this study was to investigate the effects of sex on vascular function in T2D, rather than focusing specifically on the circulating sex hormone levels.

Furthermore, we utilized a non-selective NOS inhibitor and did not assess the contributions of other NOS isoforms. Thus, the observed elevated L-NAME responses in the aortas of female ZDF rats may partly stem from increased iNOS or uncoupled eNOS. Previous studies have reported the activation of iNOS [62] and uncoupled eNOS [63] in diabetes. It has been demonstrated that the sustained increase in NO levels due to iNOS does not adequately compensate for the reduced bioavailability of NO [64]. Instead, this accumulation can be harmful, as it may react with superoxide to form peroxynitrite [64].

Lastly, while the improvement in ACh-mediated vasorelaxation following apocynin treatment, along with the increased mRNA expression of NOX4 and eNOS in the aortas of female ZDF rats, may suggest a potential role for superoxide in the impairment of EDV observed in this group; we did not measure superoxide levels. It is also important to note that our study did not assess the protein expression and activity of NOX and eNOS enzymes, and mRNA expression does not always correspond to protein levels and enzyme activity. Therefore, our findings highlight the need for further research to directly measure superoxide anion levels and evaluate the activity of various isoforms of NOX and NOS in this model. Additionally, more studies are necessary to explore the mechanisms underlying the enhanced EDV observed in male ZDF rats.

## 4. Materials and Methods

### 4.1. Materials

All chemicals were obtained from Sigma-Aldrich (St. Louis, MO, USA) and were dissolved in Milli-Q water, unless stated otherwise.

### 4.2. Experimental Animals

All animal protocols received approval from the Animal Care Committee at the University of the Pacific and adhered to the Guide for the Care and Use of Laboratory Animals: Eighth Edition [Institutional Animal Care and Use Committee (IACUC,13R07-21R01)]. The animals were euthanized using carbon dioxide (CO_2_), with the procedure following the recommendations outlined in the 2013 AVMA Guidelines on Euthanasia and the NIH Guidelines for the Care and Use of Laboratory Animals.

The ZDF rat is a well-established and widely used model for T2D, readily available from Charles River Laboratories Inc. (Wilmington, MA, USA). Male ZDF (fa/fa) rats spontaneously develop T2D on a 5008-Formulab diet (In kcal%: 26.53% protein, 56.50% carbohydrate and 16.97% fat, Labdiet, Nestlé Purina Petcare Co. St. Louis, MO, USA), while female ZDF rats require a diabetogenic diet, D12468 diet (In kcal%: 10% protein, 42.4% carbohydrate, and 47.7% fat, Research Diets Inc., New Brunswick, NJ, USA) to induce the disease [17,42,65] following the feeding/breeding instruction from Charles River. For this study, age-matched (16-week-old) rats, which were fed with the 5008-Formulab diet (for lean controls and male ZDF) or D12468 diet (for female ZDF) for 8 weeks, were divided into four groups: female lean (+/?), female ZDF (obese fa/fa), male lean (+/?), and male ZDF (obese fa/fa).

### 4.3. Measurements of Blood Glucose, Insulin, and Insulin Resistance (IR)

On the day the animals (16-week-old) were euthanized, blood was obtained by intracardiac puncture. The blood glucose level was measured instantly using a standard glucometer as reported previously [10]. Briefly, samples of blood were collected into heparinized tubes and centrifuged at 4 °C and 2000× *g* for 20 min. Plasma insulin levels were then determined by Insulin (mouse/rat) EIA Kit (#589501, Cayman Chemical, Ann Arbor, MI, USA) according to the manufacturer’s guidelines. HOMA-IR (homeostatic model assessment-insulin resistance) was calculated by using the following equation: HOMA-IR = {fasting insulin (µIU/L) x fasting glucose (nmol/L)}/22.5 [66,67]. Hancox and Landhuis [68] reported strong correlations between fasting and non-fasting blood tests for this metabolic indicator. Therefore, in the present study, we used non-fasting blood to determine insulin resistance.

### 4.4. Measurements of Aortic Wall Reactivity

We dissected and cleaned the thoracic aorta of fatty connective tissue, then sectioned it into 2 mm rings. The measurement of isometric contractile force and relaxation was carried out as previously described by us [10,12]. The ability of ACh (10 µM) to relax vessels that had been pre-contracted with phenylephrine (PE, 2 μM) served as proof of a functional endothelium.

To measure aortic relaxation to ACh (10^−8^ to 10^−5^ M) or sodium nitroprusside (SNP, 10^−9^ to 10^−5^ M, a NO donor), aortic rings were pre-contracted with a concentration of PE (2 µM) that elicited 80% of the maximal response (EC_80_) [10]. Concentration–response curves (CRC) to ACh were conducted pre- and post-treatment with apocynin (100 μM), a NADPH oxidase inhibitor, for 20 min. Aortic rings were washed with Krebs solution to maintain the basal tone between two CRC. To evaluate contractile force, CRC to PE (10^−8^ to 10^−5^ M) were obtained before and after treatment with L-NAME (200 µM), a nonselective nitric oxide synthase (NOS) inhibitor, in the presence of indomethacin (10 μM, dissolved in DMSO), a cyclooxygenase (COX) inhibitor.

The first CRC to PE (10^−8^ to 10^−5^ M) was measured in absence of any drug. The second CRC to PE was performed after treatment with indomethacin (Indo, 10 µM), which had a minor influence, and the third CRC was generated with the addition of indomethacin, followed by L-NAME (Indo, 10 µM + L-NAME, 200 µM). The data shown in this report present the second and third CRC (before and after addition of L-NAME). We also conducted a vehicle study in aortic rings of the same animals, without administering any drugs during the incubation. The first and second CRC to PE showed no significant differences.

### 4.5. Real-Time Reverse Transcription-Polymerase Chain Reaction (RT-PCR)

A real-Time RT-PCR was carried out as previously reported by us [12,13]. The primers were selected from the published literature [12,13] (The complete list of primers is shown in Table 6), and used for detection of the following gene expression: glyceraldehyde 3-phosphate dehydrogenase (GAPDH), β-actin, eNOS, NOX1, NOX2, and NOX4. Internal variations were adjusted based on rat GAPDH or β-actin, and expression levels were evaluated using the 2^−^ΔΔCt method [12,13,69]. The specificity of the PCR product was validated using agarose gel (2%) electrophoresis and ethidium bromide staining.

### 4.6. Statistical Analysis

All data are presented as mean ± standard error of the mean (SEM). Relaxation and contractile forces were calculated as previously described [12,13]. Briefly, the relaxation responses to each concentration of ACh and SNP were determined as a percentage of the maximum contraction caused by PE. The concentration of agonist that produced half of the maximum effect (E_max_) was expressed as EC_50_ and calculated by a sigmoidal dose–response model (for variable slope) using GraphPad Prism 10.3.0 (GraphPad Software Inc., San Diego, CA, USA). Sensitivity to each agonist was referred to as pD_2_ values (−log [EC_50_]), which were normally distributed. The changes in contractile force in response to increasing concentration to PE in the aortic rings were reported in grams (g), and maximum contractile force to PE was expressed as Tension_max_. The recorded increase in the force of contraction was also calculated as the percentage of maximum contraction obtained with PE at the highest dose before L-NAME. The area under the curve (AUC) was calculated using the trapezoidal method in GraphPad Prism 10.3.0. To compare the effect of PE responses under different conditions (absence/presence of L-NAME), the PE results were referred to as differences in the area under the concentration–response curve (∆AUC) between the control (absence of L-NAME) and experimental (presence of L-NAME) conditions. When the ANOVA test returned (*p* < 0.05), a post hoc analysis using Tukey’s test was performed.

A three-way ANOVA followed by Tukey’s post hoc test was used to compare differences among experimental groups in the concentration–response curve (CRC) to agonists (ACh, SNP, or PE). The three variables were as follows: (1) diabetes status (lean vs. diabetes), (2) biological sex (male vs. female), and (3) agonist concentration, which was treated as an independent repeated-measures factor.

A three-way ANOVA followed by Tukey’s post hoc test was also used to compare group means (e.g., E_max_, Tension_max_, pD_2_ in Table 3 and Table 5). The three variables were as follows: (1) diabetes status (lean vs. diabetes), (2) biological sex (male vs. female), and (3) pre-/post-inhibitor treatment.

A two-way ANOVA with repeated measures followed by Tukey’s post hoc test was used to compare CRC before and after drug treatment within a group. The two repeated-measures variables were as follows: (1) agonist concentration and (2) pre-/post-inhibitor treatment.

A two-way ANOVA followed by Tukey’s post hoc test was also used to compare group means (e.g., blood glucose levels, eNOS mRNA expression, ∆AUC, and E_max,_ Tension_max_ or pD_2_ in Table 2 and Table 4), accounting for the effects of two independent factors: (1) diabetes status (lean vs. diabetic) and (2) biological sex (male vs. female).

## Figures and Tables

**Table 1:** Body weight, non-fasting blood glucose, plasma insulin, and HOMA-IR levels in male and female lean and ZDF rats at the age of 16 weeks.

Group	*n*	Body Weight (g)	Blood Glucose (mg/dl)	Plasma Insulin (ng/mL)	HOMA-IR
Female Lean	7	202.1 ± 5.2	130.0 ± 15.7	0.80 ± 0.25	0.29 ± 0.12
Female ZDF	7	341.4 ± 5.3 ^c^	485.9 ± 26.7 ^c^	2.06 ± 0.44 (*p* = 0.06 vs. Female Lean)	2.14 ± 0.63 ^c^
Male Lean	7	365.0 ± 6.1 ^a^	135.7 ± 18.6	1.19 ± 0.07	0.40 ± 0.06
Male ZDF	7	421.0 ± 15.2 ^bd^	534.0 ± 17.9 ^d^	2.06 ± 0.43 (*p* = 0.06 vs. Male Lean)	3.00 ± 0.53 ^d^

Data are presented as mean ± SEM. ^a^ Statistical significance: *p* < 0.05 (male lean vs. female lean); ^b^ statistical significance: *p* < 0.05 (male ZDF vs. female ZDF); ^c^ statistical significance: *p* < 0.05 (female ZDF vs. female lean); ^d^ statistical significance: *p* < 0.05 (male ZDF vs. male lean), analyzed using a two-way ANOVA followed by Tukey’s post hoc test. HOMA-IR, Homeostatic Model Assessment of Insulin Resistance.

**Table 2:** pD_2_ and E_max_ values for Acetylcholine (ACh) in male and female lean and ZDF rat aortas.

Group	*n*	pD_2_	E_max_ (%)
Female lean	6	7.15 ± 0.20	83 ± 1.3
Female ZDF	6	6.97 ± 0.11	74 ± 2.0 ^c^
Male lean	6	6.69 ± 0.13	71 ± 2.5 ^a^
Male ZDF	6	7.17 ± 0.12	84 ± 1.6 ^bd^

Data are presented as mean ± SEM. ^a^ Statistical significance: *p* < 0.05 (male lean vs. female lean); ^b^ Statistical significance: *p* < 0.05 (male ZDF vs. female ZDF), ^c^ Statistical significance: *p* < 0.05 (female ZDF vs. female lean); ^d^ Statistical significance: *p* < 0.05 (male ZDF vs. male lean), analyzed using a two-way ANOVA followed by Tukey’s post hoc test. pD_2_: Sensitivity (−log EC_50_); E_max_: Maximum Response.

**Table 3:** pD_2_ and E_max_ values for Acetylcholine (ACh) before and after incubation with Apocynin (Apo) in male and female lean and ZDF rat aortas.

Group	*n*	pD_2_		E_max, %_	
		Before Apo	After Apo	Before Apo	After Apo
Female lean	6	7.25 ± 0.26	7.02 ± 0.15	81 ± 1.5	87 ± 3.1
Female ZDF	6	6.77 ± 0.16	7.02 ± 0.13	75 ± 1.6	83 ± 2.0 *
Male lean	3	7.15 ± 0.22	7.18 ± 0.18	72 ± 4.0	81 ± 1.9
Male ZDF	5	7.29 ± 0.18	7.14 ± 0.13	81 ± 3.4	86 ± 3.4

Data are presented as mean ± SEM. * Statistical significance: *p* < 0.05 (vs. before Apo in female ZDF), analyzed using a three-way ANOVA, considering Apocynin (Apo) treatment as a third independent repeated-measures factor, followed by Tukey’s post hoc test; pD_2_: Sensitivity (−log EC_50_); E_max_: Maximum Response.

**Table 4:** pD_2_ and Tension_max_ values for Phenylephrine (PE) in male and female lean and ZDF rat aortas.

Group	*n*	pD_2_	Tension_max_ (g)
Female lean	7	7.19 ± 0.09	2.39 ± 0.35
Female ZDF	7	6.97 ± 0.06	1.89 ± 0.25
Male lean	7	7.18 ± 0.06	2.39 ± 0.17
Male ZDF	7	6.89 ± 0.12	1.86 ± 0.33

Data are expressed as mean ± SEM, analyzed using a two-way ANOVA. pD_2_: Sensitivity (−logEC_50_); Tension_max_: Maximum Tension.

**Table 5:** E_max_, Tension_max_, pD_2_, and ∆AUC values for Phenylephrine (PE) before and after incubation with L-NAME in the presence of Indomethacin in male and female lean and ZDF rat aortas.

	*n*	E_max_ (%)	Tension_max_ (g)	pD_2_	∆AUC
Female lean	7				
Before		86.20 ± 2.62	2.27 ± 0.33	7.14 ± 0.09	-
After		123.46 ± 8.48 *	3.12 ± 0.28	7.82 ± 0.18 *	114.31 ± 16.99
Female ZDF	7				
Before		87.19 ± 10.16	1.42 ± 0.16	6.92 ± 0.07	-
After		174.89 ± 13.47 *	2.85 ± 0.22 *	7.28 ± 0.11 *	215.82 ± 17.26 ^c^
Male lean	6				
Before		84.37 ± 3.67	1.87 ± 0.15	7.01 ± 0.08	-
After		116.44 ± 2.45 *	2.55 ± 0.11	7.43 ± 0.10 *	91.74 ± 4.83
Male ZDF	6				
Before		77.39 ± 5.86	1.55 ± 0.28	6.89 ± 0.09	-
After		136.57 ± 9.87 *	2.54 ± 0.30 *	7.46 ± 0.15 *	129.6 ± 6.25 ^b^

Data are expressed as mean ± SEM. * Statistical significance: *p* < 0.05 (vs. before L-NAME) analyzed using a three-way ANOVA followed by Tukey’s post hoc test. ^b^ Statistical significance: *p* < 0.05 (male ZDF vs. female ZDF), ^c^ statistical significance: *p* < 0.05 (female ZDF vs. female lean), analyzed using a two-way ANOVA followed by Tukey’s post hoc test. pD_2_: Sensitivity (−logEC_50_); E_max_: Maximum Response; Tension_max_: Maximum Tension.

**Table 6:** Primers used for Real-Time RT-PCR.

Gene	Forward Primer Sequence	Reverse Primer Sequence
Rat GAPDH	5’-TGG GTG TGA ACC ACA AGA AA-3’	5’-GTG GCA GTG ATG ACA TGG AC-3’
Rat β-actin	5’-CTG GGT ATG GAA TCC TGT GG-3’	5’-TCA TCG TAC TCC TGC TTG CTG-3’
Rat eNOS	5’- ACT GCG TCG CTT CAT TAG GT-3’	5’- TAG GCA AGC GCT TTA CCA CT-3’
Rat NOX1	5’- GGC AAC ATG AGA GCT GCA TA-3’	5’- GCA AGT GTC AAC CAG CAA GA-3’
Rat NOX2	5’-ACC CTT TCA CCCTGA CCT CT-3’	5’-TCC CAG CTC CCA CTA ACA TC-3’
Rat NOX4	5’-CCA GAA TGA GGA TCC CAGAA-3’	5’-AGC AGC AGC AGC ATG TAG AA-3’

GAPDH, Glyceraldehyde 3-phosphate dehydrogenase; β-actin, beta actin; eNOS, endothelial nitric oxide; NOX1, NADPH oxidase 1; NOX2, NADPH oxidase 2; NOX4, NADPH oxidase 4.

## Data Availability

The original contributions outlined in the study are incorporated within the article.
